# *Toxoplasma gondii* Profilin Promotes Recruitment of Ly6C^hi^ CCR2^+^ Inflammatory Monocytes That Can Confer Resistance to Bacterial Infection

**DOI:** 10.1371/journal.ppat.1004203

**Published:** 2014-06-12

**Authors:** Lori M. Neal, Laura J. Knoll

**Affiliations:** Department of Medical Microbiology and Immunology, University of Wisconsin - Madison, Madison, Wisconsin, United States of America; University of Michigan Medical School, United States of America

## Abstract

Ly6C^+^ inflammatory monocytes are essential to host defense against *Toxoplasma gondii, Listeria monocytogenes* and other infections. During *T. gondii* infection impaired inflammatory monocyte emigration results in severe inflammation and failure to control parasite replication. However, the *T. gondii* factors that elicit these monocytes are unknown. Early studies from the Remington laboratory showed that mice with a chronic *T. gondii* infection survive lethal co-infections with unrelated pathogens, including *L*. *monocytogenes*, but a mechanistic analysis was not performed. Here we report that this enhanced survival against *L. monocytogenes* is due to early reduction of bacterial burdens and elicitation of Ly6C^+^ inflammatory monocytes. We demonstrate that a single TLR11/TLR12 ligand profilin (TgPRF) was sufficient to reduce bacterial burdens similar to *T. gondii* chronic infection. Stimulation with TgPRF was also sufficient to enhance animal survival when administered either pre- or post-*Listeria* infection. The ability of TgPRF to reduce *L. monocytogenes* burdens was dependent on TLR11 and required IFN-γ but was not dependent on IL-12 signaling. TgPRF induced rapid production of MCP-1 and resulted in trafficking of Ly6C^hi^ CCR2^+^ inflammatory monocytes and Ly6G^+^ neutrophils into the blood and spleen. Stimulation with TgPRF reduced *L. monocytogenes* burdens in mice depleted with the Ly6G specific MAb 1A8, but not in Ly6C/Ly6G specific RB6-8C5 depleted or CCR2^−/−^ mice, indicating that only inflammatory monocytes are required for TgPRF-induced reduction in bacterial burdens. These results demonstrate that stimulation of TLR11 by TgPRF is a mechanism to promote the emigration of Ly6C^hi^ CCR2^+^ monocytes, and that TgPRF recruited inflammatory monocytes can provide an immunological benefit against an unrelated pathogen.

## Author Summary

*Toxoplasma gondii* is an apicomplexan parasite that can infect all warm blooded animals, but rodent species are considered the primary reservoirs. Mice that are infected with *T. gondii* become more resistant to lethal infection with other pathogens. Ly6C^+^ inflammatory monocytes are innate immune cells that are critical for defense against *T. gondii* and other infections. Mice with defects in the ability to recruit inflammatory monocytes fail to control *T. gondii* replication and succumb to overwhelming inflammation. In this study we used a co-infection model to explain why *T. gondii*-infected mice are more resistant to the bacterium *Listeria monocytogenes*. We show that stimulation of the rodent specific Toll-like receptor TLR11 by the *T. gondii* ligand profilin can recruit inflammatory monocytes, and that these monocytes can protect the host against *L. monocytogenes*. These findings make profilin an important tool for the study of monocyte biology during *T. gondii* infection of rodents and are especially interesting given that TLR11 is nonfunctional in humans and other vertebrates.

## Introduction

*Toxoplasma gondii* is an obligate intracellular Apicomplexan parasite that can infect nearly any nucleated cell of all warm blooded animals. Within warm blooded hosts, *T. gondii* replicates as a fast growing tachyzoite form, which disseminates throughout the body during acute infection. Over time and under immune pressure, the parasite differentiates into an encysted bradyzoite form within the central nervous system and muscle tissue, which establishes a life-long chronic infection. Approximately 30% of humans are infected with *T. gondii* but the infection may be asymptomatic in immunocompetent hosts.

*T. gondii* infection is characterized by a highly polarized Th1 type immune response associated with production of IL-12 by dendritic cells (DCs), neutrophils, and macrophages which drives T and NK cell production of IFN-γ, long regarded as the main mediator of acute and chronic defenses against the parasite [1], [2], [3]. One of the *T. gondii* proteins known to stimulate IL-12 production is *T. gondii* profilin (TgPRF), which is required for parasite actin remodeling during host cell invasion and egress, and is also a ligand for TLR11 and TLR12 [4], [5], [6], [7]. Another critical factor for innate defenses are a class of Gr-1^+^ Ly6C^+^ monocytes that produce nitric oxide (NO) and TNF-α, and are recruited in a CCR2 dependent manner in response to both oral and parenteral *T. gondii* infections [8], [9], [10], [11]. MCP-1^−/−^ and CCR2^−/−^ mice do not recruit Ly6C^+^ monocytes to the lamina propria in response to oral infection, leading to a higher influx of neutrophils and death from intestinal necrosis and inflammation [8], [9]. Similarly, MCP-1^−/−^ and CCR2^−/−^ mice fail to recruit inflammatory monocytes to the peritoneal cavity following i.p. inoculation leading to increased mortality and parasite burdens [10]. Thus, Ly6C^+^ monocytes are necessary for early control of *T. gondii* replication and to prevent immune pathology. However, the specific parasite factors that elicit Ly6C^+^ monocytes during *T. gondii* infection have not been identified. Ly6C^hi^ monocytes are also recruited during infections with other protozoan and bacterial pathogens, including *Listeria monocytogenes* [12], [13], [14], [15], [16].

*T. gondii* sexual reproduction occurs exclusively in the intestines of the feline definitive hosts, making the rodents they prey on key intermediate hosts in the *T. gondii* lifecycle. *T. gondii* infection has been shown to alter rodent aversion to cat urine and fear avoidance behaviors in ways that increase the odds of predation and thus parasite reproductive success [17], [18]. Previous studies have also reported that mice infected with *T. gondii* are more resistant to secondary infections with unrelated pathogens, including *L. monocytogenes, Salmonella typhimurium*, mengo virus, *Cryptococcus neoformans, Besnoita jejuni*, Moloney leukemia virus and *Schistosoma monsoni* [19], [20], [21], [22], [23], [24], which may also serve to increase predation. We have recently shown that stimulation with soluble *T. gondii* antigens (STAg) reduced viral titers and conferred a survival advantage in mice infected with highly pathogenic H5N1 avian influenza virus [25], demonstrating that treatment with STAg can stimulate immunity against unrelated pathogens. In order to further investigate the mechanisms conferring this immunological benefit, we used a highly tractable *L. monocytogenes* infection model.

*L. monocytogenes* is a Gram positive facultative intracellular bacteria commonly associated with outbreaks of the foodborne illness listeriosis. In mice, intravenous inoculation with *L. monocytogenes* causes highly predictable infection, involving both innate and adaptive immune responses that ultimately clear the bacteria [26], [27]. Before the onset of adaptive immunity, bacteria replicate primarily in infectious foci within cells of the spleen and liver where innate immune responses are critical for controlling early bacterial growth to prevent dissemination and lethal systemic infection. Increased early bacterial burdens in the spleen and liver correlate with the severity and outcome of infection.

Ly6C^hi^ CCR2^+^ inflammatory monocytes mediate critical innate control of early bacterial replication. During *L. monocytogenes* infection, Ly6C^hi^ CCR2^+^ cells emigrate from the bone marrow in a CCR2-dependent manor, and traffic to sites of bacterial infection to differentiate into CD11C^+^ TNF-α and inducible nitric oxide synthase (iNOS) producing DCs (TipDCs) that enhance bacterial clearance [12], [15], [28]. Emigration of Ly6C^hi^ CCR2^+^ cells from the bone marrow is directed by MCP-1 and MCP-3, which is mainly produced by non-hematopoietic cells during infection and can be produced by bone marrow mesenchymal stem cells (BMSCs) in response to circulating TLR ligands [12], [16], [29], [30]. Accordingly, CCR2^−/−^ mice have reduced numbers of circulating Ly6C^hi^ monocytes, reduced numbers of TipDCs in the spleen and liver, reduced TNF-α production and are more susceptible to *L. monocytogenes* infection [12], [15], [16], [28]. IFN-γ and TNF-α are essential to the innate response as mice lacking either cytokine rapidly succumb to *L. monocytogenes* infection [31], [32], [33].

In this study we show that chronic *T. gondii* infection or stimulation with STAg provides resistance against *L. monocytogenes* bacterial infection by reducing bacterial burdens in the major sites of bacterial replication, the spleen and liver. We also show that stimulation with the TgPRF is sufficient to induce this resistance independent of IL-12, T and NK1.1^+^ cells but cannot completely overcome the requirement for IFN-γ mediated defenses. Most importantly, we show that TgPRF induces production of MCP-1, which results in the trafficking of Ly6C^hi^ CCR2^+^ inflammatory monocytes into the blood and spleen, and that CCR2-dependent recruitment of these cells is essential to the TgPRF-induced anti-bacterial response. These results demonstrate that stimulation of TLR11 by TgPRF is sufficient to promote recruitment of Ly6C^hi^ CCR2^+^ inflammatory monocytes, and that these monocytes can provide and immunological benefit against other infections.

## Results

### Chronic infection with *T. gondii* confers resistance against *L. monocytogenes* infection

Previous research has shown that mice with a chronic *T. gondii* infection had greater survival or delayed time to death when challenged with a lethal *L. monocytogenes* infection [19]. Further experiments showed that this protective effect was not transferrable in the serum, and thus was likely a cell mediated response [34]. Although the specific bacterial burdens were not determined for the animals in these studies, early innate control of *L. monocytogenes* replication correlates well with severity of infection in mice: animals that maintain low bacterial numbers generally go on to clear the infection, whereas failure of innate immunity is associated with high numbers of bacteria, overwhelming sepsis and inevitable death. We hypothesized that the enhanced survival of *T. gondii* infected mice was due to innate control of *L. monocytogenes* replication.

**Figure 1:**
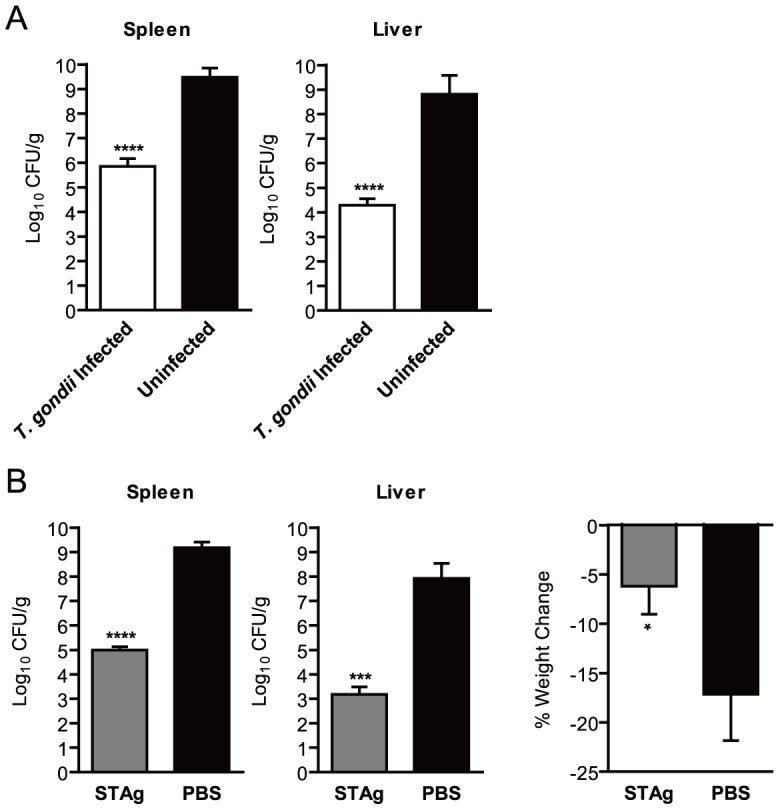
Chronic *T. gondii* infection or stimulation with STAg reduces bacterial burden during *L. monocytogenes* infection. (A) Mice (n = 6/group) were infected i.p. with 250 *T. gondii* tachyzoites and allowed to establish a chronic infection (white) or kept uninfected in the same facility as age-matched controls (black). On day 36, the mice were infected with ∼6×10^4^ CFU of *L. monocytogenes*. At 72 hours post *L. monocytogenes* infection, the number of CFU per gram of spleen or liver (CFU/g) was determined. One uninfected mouse died of *L. monocytogenes* infection prior to the time when bacterial burden was assessed and was assigned a burden of 8.0 log_10_(CFU/g) for statistical purposes. Data shown are the mean ± SD from one of two independent experiments. (B) Naïve mice (n = 3/group) were stimulated i.v. with 1 µl of STAg (grey) or PBS (black) 24 hours prior infection with ∼6×10^4^ CFU of *L. monocytogenes*. At 72 hours post *L. monocytogenes* infection, bacterial burdens of the spleen and liver, and percent weight change as compared to immediately prior to infection were quantified. Data shown are the mean ± SD from one of more than ten independent experiments. (A–B) *** indicates p<0.001 and **** indicates p<0.0001.

To test this hypothesis, we infected naïve and *T. gondii* chronically infected mice with a lethal inoculum of *L. monocytogenes,* and then we determined the number of viable bacteria in the spleens and livers 72 hours later. *T. gondii*-infected mice had significant ∼3.6 log reductions in bacterial burdens in the spleen ∼4.5 log reductions in the liver compared to uninfected controls (Fig. 1A). In our experience, mice with bacterial burden less than 6 log_10_ CFU/g in the spleen and liver at 72 hours post infection typically remain asymptomatic and survive *L. monocytogenes* infection; whereas those with higher bacterial burdens usually succumb to infection. As the bacterial burdens in *T. gondii* infected mice were consistently less than 6 log_10_ CFU/g in both organs (Fig. 1A), these results suggest that survival of *T. gondii* infected mice reported previously [19] was due to early reductions in the numbers or replication of *L. monocytogenes* bacteria.

### Stimulation with STAg reduces bacterial burden following *L. monocytogenes* infection

Our previous work with influenza virus [25] had shown that the protective effects of *T. gondii* infection could be replicated by treating mice with STAg, a non-infectious lysate of soluble antigens from sonicated *T. gondii* tachyzoites. STAg contains many *T. gondii* proteins, including profilin [5], and previous work has shown that STAg can stimulate immune responses similar to those induced by live parasites, including induction of IL-12, TNF-α, IFN-γ, IL-1β, IL-10 and MCP-1 *in vivo* or *in vitro* [35], [36], [37]. Consistent with these data, we observed increased levels of IL-12, TNF-α, IFN-γ and MCP-1 in the serum of STAg-stimulated mice within 24 hours (data not shown). We hypothesized that STAg treatment would reduce the bacterial burdens of *L. monocytogenes* infected mice as well as chronic *T. gondii* infection. Mice treated with 1 µl of STAg (approximately 1 µg total protein) 24 hours prior to infection with *L. monocytogenes* had ∼2.5 log reductions in bacterial burden in the spleens and ∼3.8 log reductions in the liver compared to PBS-treated controls (Fig. 1B). These effects were similar to the reduction in bacterial burdens we observed in *T. gondii* infected mice (Fig. 1A). STAg stimulated mice also experienced significantly less weight loss than PBS treated controls at 72 hours post infection (Fig. 1B). STAg stimulation was effective for reducing bacterial burdens and weight loss when given 2 or 6 hours post *L. monocytogenes* infection, although the reduction in bacterial burdens began to decline at 6 hours (data not shown).

To determine if the protective components in STAg were protein or other molecules such as RNA or DNA, we subjected STAg to proteinase K digestion. Proteinase K-digested STAg did not reduce bacterial burdens in *L. monocytogenes* infected mice (Fig. S1A), which suggested that the protective component(s) were protein. To identify the specific protein(s), we subjected STAg to ammonium sulfate (AS) precipitation and assayed the fractions for their ability to reduce the bacterial burdens. The AS precipitation fraction containing the proteins that remained soluble at AS concentrations >60% reduced the bacterial burdens similar to STAg (Fig. S1B). When we subjected these fractions to western blotting with antibodies against several *T. gondii* proteins, we saw TgPRF was present in the AS >60% fraction (Fig. S1C).

### Recombinant TgPRF is sufficient to reduce bacterial burden and enhance survival following *L. monocytogenes* infection

**Figure 2:**
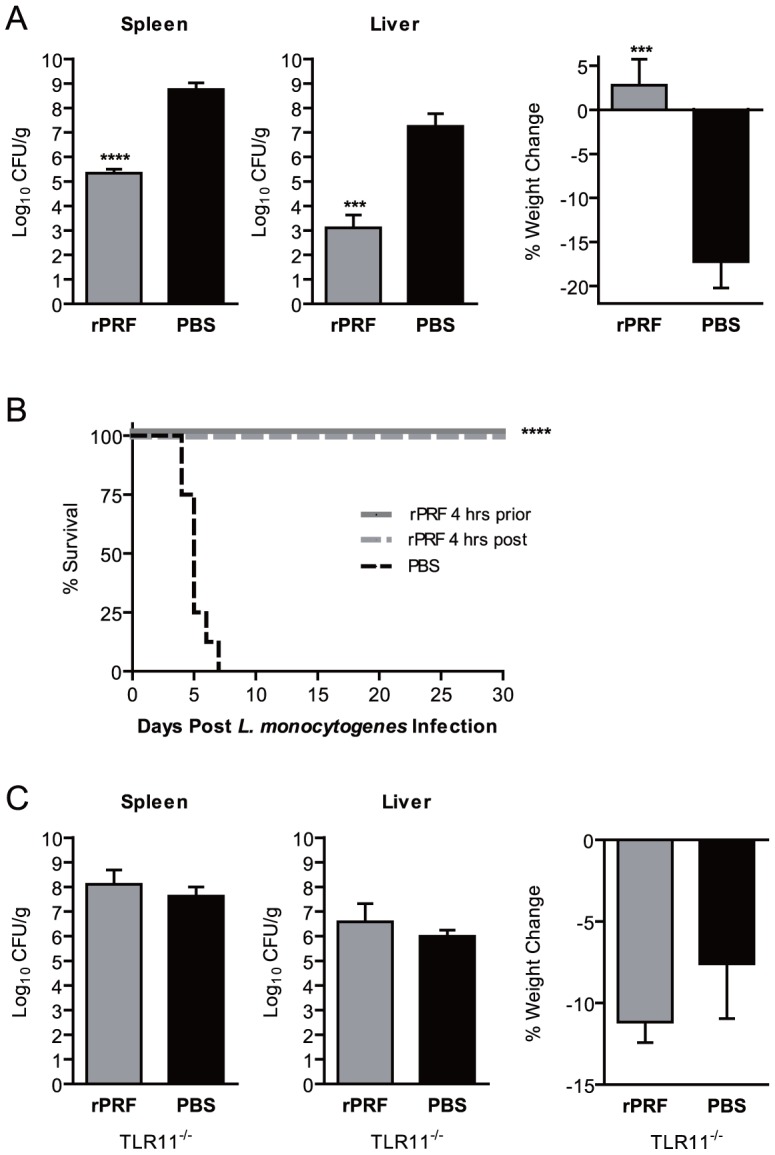
Stimulation with *T. gondii* profilin is sufficient to confer resistance to *L. monocytogenes*. (A) Mice (n = 3/group) were stimulated i.v. with 100 ng of recombinant *T. gondii* profilin (rPRF) (grey) or PBS (black) 4 hours prior infection with ∼6×10^4^ CFU of *L. monocytogenes*. At 72 hours post *L. monocytogenes* infection, bacterial burdens of the spleen and liver, and percent weight change as compared to immediately prior to infection were quantified. Data shown are the mean ± SD from one of five independent experiments. *** indicates p<0.001 and **** indicates p<0.0001. (B) Survival of *L. monocytogenes* infected mice (n = 8/group) stimulated with PBS (dotted black) or rPRF 4 hours prior (solid grey) or post (dotted grey) *L. monocytogenes* infection (p<0.0001). (C) TLR11-deficient (TLR11^−/−^) mice (n = 4/group) were stimulated i.v. with 4 µg of recombinant rPRF (grey) or PBS (black) 4 hours prior infection with ∼4×10^4^ CFU of *L. monocytogenes*. At 72 hours post *L. monocytogenes* infection, bacterial burdens of the spleen and liver, and percent weight change as compared to immediately prior to infection were quantified. Data shown are the mean ± SD from one of four independent experiments.

TgPRF is an actin-binding protein involved in parasite gliding motility, host cell invasion and egress, and is known for inducing IL-12 production through stimulation of TLR11 and TLR12 expressed on DCs and macrophages [4], [5], [6]. In order to determine if TgPRF was sufficient to confer protection against *L. monocytogenes* we stimulated mice with purified recombinant N-terminal his-tagged TgPRF (rPRF) (Fig. 2A). Mice stimulated with 100 ng rPRF 4 hours prior to *L. monocytogenes* infection had a significant ∼3.4 log reduction in bacterial burdens in the spleen and ∼4 log reduction in the liver compared to PBS-treated animals (Fig. 2A). rPRF-treated mice did not exhibit weight loss in contrast to PBS-treated controls which lost 17% of their starting weight by 72 hours post infection (Fig. 2A).

Because stimulation with rPRF was sufficient to reduce bacterial burdens similar to *T. gondii* infection (Fig. 1A), we expected rPRF to enhance survival of *L. monocytogenes* infected mice in our model (Fig. 2B). All (8/8) PBS-treated mice rapidly succumbed to *L. monocytogenes* infection within 7 days, with the majority of mice succumbing by day 5. In contrast, 100% (8/8 for each group) of mice stimulated with rPRF 4 hours prior to, or 4 hours after, *L. monocytogenes* infection survived for 30 days, at which point the experiment was terminated. These results demonstrate that rPRF-stimulation is sufficient to reduce bacterial burdens and confer a long-term survival advantage during *L. monocytogenes* infection.

Although TgPRF can be recognized by TLR11 and TLR12 [5], [6], [7], the ability of rPRF to reduce bacterial burdens was strictly dependent on TLR11. In multiple experiments, TLR11-deficient (TLR11^−/−^) mice treated with 40-fold more protein (4 µg rPRF) 4 hours prior to *L. monocytogenes* infection had no reduction in bacterial burden in either the spleen or liver compared to PBS-stimulated controls (Fig. 2C). rPRF-stimulated TLR11^−/−^ mice also showed equivalent weight loss as the PBS controls (Fig. 2C). These results demonstrate that the effects of rPRF are dependent on recognition by TLR11 and that potential contaminants such as LPS do not contribute to the effect.

To determine whether TgPRF was the major *T. gondii* factor in STAg responsible for the resistance to *L. monocytogenes,* we stimulated TLR11^−/−^ mice with STAg. Doses of STAg up to 200 µl did not result in significant reductions in bacterial burdens or reduced weight loss during *L. monocytogenes* infection (Fig. S2 and data not shown). We did observe modest but statistically significant reductions in bacterial burdens in the spleen (∼20-fold) and liver (∼80-fold) with 200 µl of STAg generated from twice the normal number of parasites, or 400 µg of protein (Fig. S2). These results were in contrast to WT mice, in which 1 µg of STAg reduced bacterial burdens by up 300-fold in the spleen and 6,000-fold in the liver (Fig. 1B). It is possible that the TLR11 independent effects of STAg could be due to parasite derived TLR ligands such as nucleic acids and GPI moieties, or other parasite derived proteins. However, it is unlikely that such large doses of STAg, equivalent to material from 1.6×10^8^ lysed parasites, are relevant during natural infection and thus TgPRF is likely to be the main factor in STAg responsible for the resistance to *L. monocytogenes*.

### TgPRF induces production of IL-12, IFN-γ, MCP-1 and TNF-α

**Figure 3:**
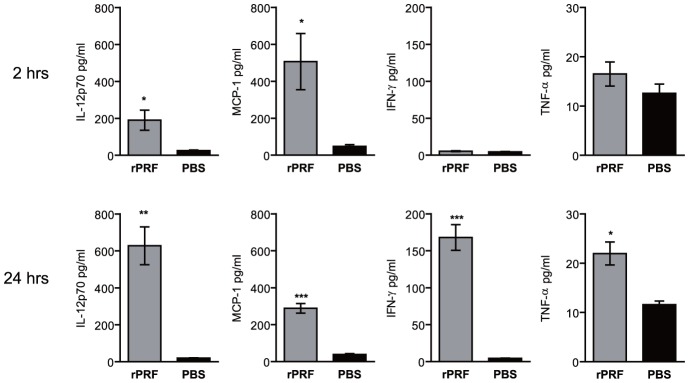
*T. gondii* profilin induces production of IL-12, MCP-1, IFN-γ, and TNF-α. Mice (n = 3-4/group) were stimulated i.v. with 100 ng rPRF (grey) or PBS (black). Serum was collected 2 or 24 hours later and assayed for cytokine levels by cytometric bead array. Data shown are the mean ± SEM from one of two independent experiments. * indicates p<0.05, ** indicates p<0.01, and *** indicates p<0.001.

STAg has been shown to induce cytokines and chemokines including IL-12, TNF-α, IFN-γ, IL-1β, IL-10 and MCP-1 [35], [36], [37]. TgPRF has been shown to induce IL-12 by classes of DCs and macrophages, IFN-α by CD11c^+^ spleenocytes, and to promote IFN-γ production by NK1.1^+^ cells [6]. To determine if TgPRF could induce production of other anti-listerial cytokines, we stimulated mice with rPRF then analyzed serum 2 or 24 hours later. rPRF stimulation induced significant production of IL-12 and MCP-1 at 2 and 24 hours, and IFN-γ and TNF-α by 24 hours (Fig. 3). These results show that TgPRF can stimulate the production of multiple cytokines and chemokines in addition to IL-12.

### Signaling through IL-12Rβ1 is not required for TgPRF-induction reduction in bacterial burden

**Figure 4:**
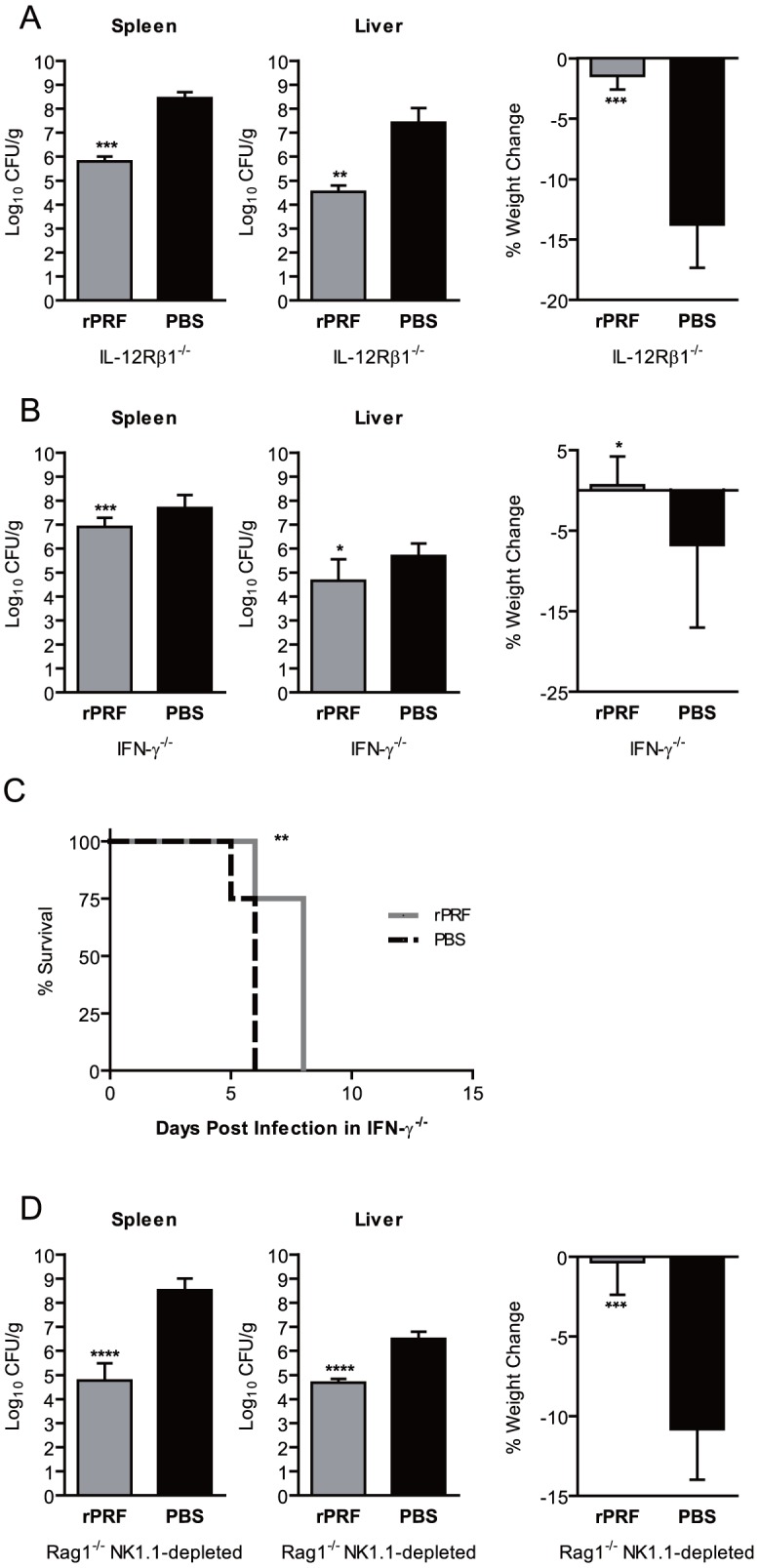
*T. gondii* profilin induced protection against *L. monocytogenes* is independent of IL-12 and T and NK cells, but dependent on IFN-γ. (A) IL-12Rβ1 deficient (IL-12Rβ1^−/−^) mice (n = 4–5/group) were stimulated i.v. with 100 ng rPRF (grey) or PBS (black) 4 hours prior infection with ∼1×10^4^ CFU of *L. monocytogenes*. At 72 hours post *L. monocytogenes* infection, bacterial burdens of the spleen and liver, and percent weight change as compared to immediately prior to infection were quantified. Data shown are the mean ± SD from one of four independent experiments. (B) IFN-γ deficient (IFN-γ ^−/−^) mice were stimulated i.v. with 100 ng rPRF (grey) or PBS (black) 4 or 24 hours prior to infection with ∼200 CFU of *L. monocytogenes*. Bacterial burdens of the spleen and liver, and percent weight change as compared to immediately prior to infection were quantified after 72 hours. Data shown are the mean ± SD from three experiments (each n = 3–7/group). (C) Survival of IFN-γ ^−/−^ mice (n = 6/group) stimulated with 100 ng rPRF (grey) or PBS (black) 4 hours prior to infection with ∼200 CFU of *L. monocytogenes*. (D) Rag1 deficient (Rag1^−/−^) mice depleted with anti-NK1.1 MAb PK136 (n = 4/group) were stimulated i.v. with 100 ng rPRF (grey) or PBS (black) 4 hours prior infection with ∼8×10^4^ CFU of *L. monocytogenes*. At 72 hours post *L. monocytogenes* infection, bacterial burdens of the spleen and liver, and percent weight change as compared to immediately prior to infection were quantified. Data shown are the mean ± SD from one of two independent experiments. (A–D) * indicates p<0.05, ** indicates p<0.01, *** indicates p<0.001, and **** indicates p<0.0001.

IL-12 mediates defenses against *T. gondii* by inducing IFN-γ production from NK and T cells, which in turn helps to activate macrophage effector functions, enhancing antigen presentation, and by promoting the differentiation of Th1 cells [2]. IL-12 plays a similar and critical role in *L. monocytogenes* infections [26], [27]. We hypothesized that the ability of rPRF to reduce the bacterial burdens would require IL-12 signaling. However, we determined that IL-12 signaling was not required for rPRF-induced resistance to *L. monocytogenes* infection using IL-12Rβ1 deficient (IL-12Rβ1^−/−^) mice (Fig. 4A). Compared to PBS-treated controls, rPRF-treated IL-12Rβ1^−/−^ mice had significant ∼2.6 log and ∼2.8 log reductions in bacterial burdens in the spleen and livers, respectively. rPRF-treated IL-12Rβ1^−/−^ mice exhibited only mild weight loss of 1.5%, in contrast to PBS-treated controls which lost 14% of their weight. IL-12Rβ1 is also a component of the IL-23 receptor, so these results indicate that both IL-12 and IL-23 signaling are not required for rPRF-induced resistance to *L. monocytogenes* infection.

### IFN-γ but not T and NK1.1^+^ cells are required for TgPRF induced protection

IFN-γ is a critical mediator of innate defenses against both *L. monocytogenes* [31], [32] and *T. gondii* [1], [3]. Our previous work with STAg and influenza virus found that STAg-induced IFN-γ from NK cells was required to mediate protection against influenza virus [25]. To determine the role of IFN-γ mediated defenses in rPRF-induced protection against *L. monocytogenes* we treated IFN-γ deficient (IFN-γ^−/−^) mice with rPRF (Fig. 4B) then infected them with a low but lethal dose of *L. monocytogenes*, 200 CFU/animal, to account for the extreme susceptibility imposed by IFN-γ deficiency [31], [32]. rPRF-treated IFN-γ^−/−^ mice had a slight but statistically significant 6-fold reduction in bacterial burden in the spleen and 10-fold reduction in the liver compared to PBS-treated controls. Although the bacterial burdens were still high, rPRF-treated IFN-γ^−/−^ mice experienced less weight loss than PBS-treated controls. While all rPRF-treated IFN-γ^−/−^ mice did succumb to *L. monocytogenes* infection within eight days, the delay was significant relative to PBS-stimulated animals (Fig. 4C). These results suggest that IFN-γ is at least partially required for rPRF-induced protection against *L. monocytogenes* and that rPRF stimulation cannot overcome the requirement for IFN-γ mediated defenses even at the low infectious doses used.

The major sources of IFN-γ are T cells and NK cells. NK1.1^+^ cells are the critical source of IFN-γ for early defense against *T. gondii* [38] and for STAg-induced protection against influenza virus [25]. Similarly, the majority of IFN-γ during early *L. monocytogenes* infection is produced by NK1.1^+^ cells [39], [40]. However, T cells can also produce IFN-γ in early responses to *T. gondii* [41] and *L. monocytogenes* infection [40], [42]. To determine if either NK1.1^+^ or T cells were required for rPRF-induced for protection against *L. monocytogenes*, we created mice deficient in both T and NK cells by depleting Rag1 deficient (Rag1^−/−^) mice with PK136 (anti-NK1.1) monoclonal antibody. In contrast to IFN-γ^−/−^ mice, Rag1^−/−^ NK1.1-depleted mice had no increase in susceptibility to *L. monocytogenes* infection and rPRF-stimulation was highly effective in Rag1^−/−^ NK1.1-depleted mice infected with 6×10^4^ CFU/animal, the same dose used for experiments with WT animals (Fig. 4D). rPRF-treated mice had a ∼3.7 log reduction in bacterial burden in the spleen and ∼1.8 log reduction in the liver, compared to PBS-treated controls. rPRF-treated Rag1^−/−^ NK1.1-depleted mice also did not show weight loss (Fig. 4D). Similar results were observed with rPRF treatment in singly deficient Rag1^−/−^ or wild-type NK1.1-depleted mice (data not shown). These data suggest that neither T nor NK cells are required for rPRF-induced reduction in bacterial burdens and survival. However, in the absence of T and NK cells, mice may develop compensatory defense mechanisms, so it is possible the factors required in these animals are different than in WT mice.

### Ly6C^hi^ CCR2^+^ inflammatory monocytes and Ly6C^int^ Ly6G^+^ neutrophils are recruited in response to TgPRF

**Figure 5:**
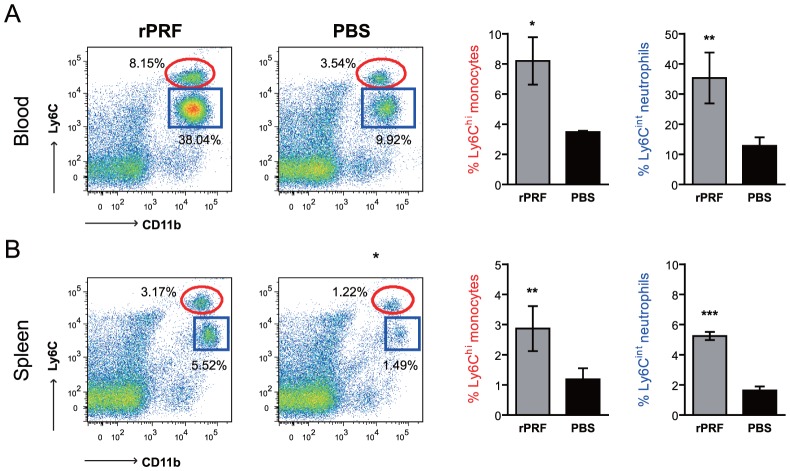
Ly6C^hi^ CCR2^+^ inflammatory monocytes and Ly6C^int^ Ly6G^+^ neutrophils are recruited to the blood and spleen in response to *T. gondii* profilin. Mice (n = 3–4/group) were stimulated i.v. with 100 ng rPRF (grey) or PBS (black) then blood (A) and spleen (B) cells were collected 4 hours later and analyzed for expression of CD45, CD11b, Ly6C, Ly6G, and CCR2 by flow cytometry. Gating and analysis was conducted on singlet, live, CD45^+^ cells. CD11b^+^ Ly6C^hi^ monocytes are within the red elliptical gates and were CCR2^+^ Ly6G^−^. Neutrophils are shown in the blue square gates and were CCR2^−^ Ly6G^+^. The data shown are from one experiment, representative of at least 5 independent experiments. One data point from the PBS group was a statistical outlier, as calculated by Grubb's test, and was excluded from analysis. * indicates p<0.05, ** indicates p<0.01, *** indicates p<0.001.

During *L. monocytogenes* infection, MCP-1 and MCP-3 signals promote emigration of TipDC precursors, Ly6C^hi^ inflammatory monocytes, out of the bone marrow and into circulation in a CCR2-dependent manner [16]. Because serum levels of MCP-1 in rPRF-stimulated mice were significantly increased within 2 hours (Fig. 3) and because *T. gondii* infection is also known to elicit a population of Ly6C^+^ monocytes via CCR2 [8], [9], [10], we examined the ability of rPRF to promote emigration of Ly6C^hi^ monocytes. Within four hours after rPRF stimulation, there was an ∼3 fold average increase in the frequency of CD11b^+^ Ly6C^hi^ monocytes in both the blood and spleens of TgPRF stimulated animals (Fig. 5A and B).The Ly6C^hi^ monocyte population expressed CCR2 (data not shown), consistent with an inflammatory monocyte and TipDC precursor populations described previously [9], [10], [11], [12], [13], [14], [15]. There was also an ∼2.7 fold average increase in the frequency of neutrophils (CD11b^+^ Ly6C^int^ Ly6G^+^) in the blood and a ∼2.5 fold average increase in the spleens of rPRF stimulated mice (Fig. 5A and B). To confirm that these results were specifically attributable to TgPRF, we measured monocyte and neutrophil recruitment in TLR11^−/−^ mice. As expected, there was not an increase the percentage of Ly6C^hi^ monocytes or neutrophils in TLR11^−/−^ mice stimulated with 100 ng rPRF compared to PBS stimulated controls (Fig. S3), demonstrating the specificity of the TgPRF-TLR11 interaction in monocyte and neutrophil recruitment.

### Ly6C^hi^ CCR2^+^ inflammatory monocytes are required for TgPRF-induced defenses

**Figure 6:**
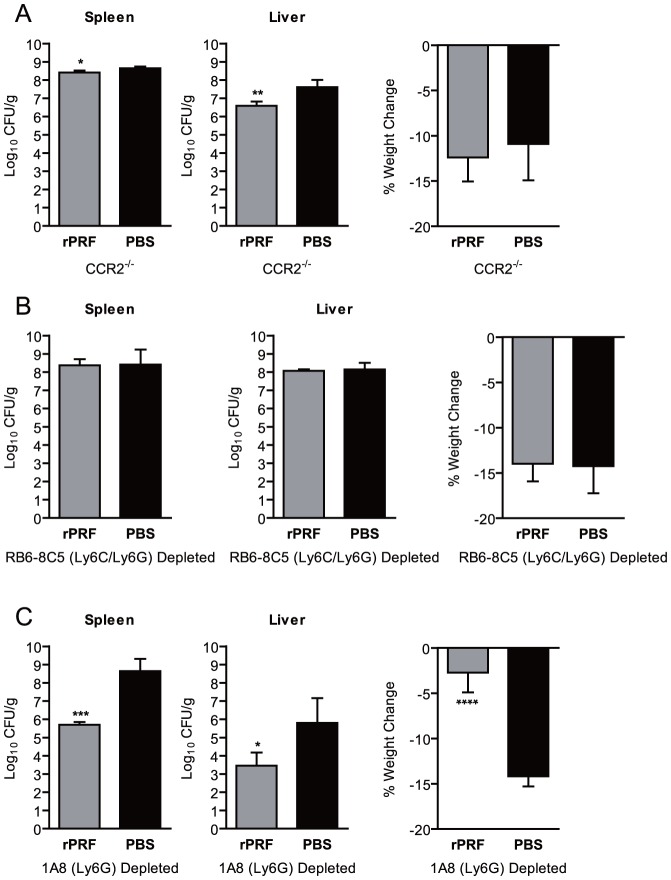
CCR2-dependent recruitment of Ly6C^+^ cells, but not recruitment of Ly6G^+^ cells, is essential for *T. gondii* profilin-induced protection against *L. monocytogenes*. (A) CCR2 deficient (CCR2^−/−^) mice (n = 4–5/group) were stimulated i.v. with 100 ng rPRF (grey) or PBS (black) 4 hours prior infection with ∼8×10^3^ CFU of *L. monocytogenes.* At 72 hours post *L. monocytogenes* infection, bacterial burdens of the spleen and liver, and percent weight change as compared to immediately prior to infection were quantified. Data shown are the mean ± SD from one of six independent experiments. (B) Mice depleted with anti-Gr-1 (Ly6c/Ly6G) MAb RB6-8C5 (n = 4/group) were stimulated i.v. with 100 ng rPRF (grey) or PBS (black) 4 hours prior infection with ∼200 CFU of *L. monocytogenes.* At 72 hours post *L. monocytogenes* infection, bacterial burdens of the spleen and liver, and percent weight change as compared to immediately prior to infection were quantified. Data shown are the mean ± SD from one of three independent experiments. (C) Mice depleted with anti-Ly6G MAb 1A8 (n = 4/group) were stimulated i.v. with 100 ng rPRF (grey) or PBS (black) 4 hours prior infection with ∼8×10^3^ CFU of *L. monocytogenes*. At 72 hours post *L. monocytogenes* infection, bacterial burdens of the spleen and liver, and percent weight change as compared to immediately prior to infection were quantified. Data shown are the mean ± SD from one of three independent experiments. (A–C) * indicates p<0.05, ** indicates p<0.01, *** indicates p<0.001, and **** indicates p<0.0001.

Ly6C^hi^ CCR2^+^ monocyte emigration from the bone marrow into circulation is CCR2-dependent [9], [12]. To determine if Ly6C^hi^ CCR2^+^ cells recruited in response to rPRF were essential for the reductions in bacterial burdens, we rPRF-stimulated CCR2 deficient (CCR2^−/−^) mice (Fig. 6A). rPRF-stimulated CCR2^−/−^ mice did not have large reductions in bacterial burdens compared to PBS-treated controls, 2-fold in the spleen and 10-fold in the liver. Although the reductions were statistically significant, they are not likely biologically relevant given the overall high burdens. In addition, both groups experienced equal weight loss.

Although CCR2^−/−^ mice have diminished levels of circulating Ly6C^hi^ monocytes, they have increased numbers in the bone marrow at rest, and large numbers of activated TNF-α producing Ly6C^hi^ monocytes accumulate in the bone marrow during infection [12]. Thus, the small reduction in bacterial burden we saw in rPRF-stimulated CCR2^−/−^ mice could still be dependent on Ly6C^hi^ CCR2^+^ monocytes, either by activation of a limited number of cells in circulation, or via soluble cytokines such as TNF-α produced by those cells restricted to the bone marrow. To deplete Ly6C^hi^ CCR2^+^ monocytes, we treated mice with the anti-Gr-1 MAb RB6-8C5, which recognizes a common epitope shared by Ly6C and Ly6G [43]. Depletion with MAb RB6-8C5 reduced neutrophils in the spleens of rPRF stimulated mice by ∼95% and inflammatory monocytes by ∼85% (data not shown). We consistently observed that rPRF-stimulation did not offer any protection in RB6-8C5 depleted mice. There were no significant difference in bacterial burdens between rPRF- and PBS-stimulated mice in either the spleens or livers (Fig. 6B), and both groups experienced equal weight loss (Fig. 6B). Because TLR11 and TLR12 are expressed on macrophages and DCs [7], which may express Ly6C and thus would be depleted by RB6-8C5, we tested the ability of RB6-8C5 depleted mice to respond to profilin by measuring serum cytokine levels 2 hours post rPRF stimulation. rPRF-stimulated RB6-8C5 depleted mice produced significant amounts of IL-12 and MCP-1 (Fig. S4) at levels similar to WT mice at the same timepoint (Fig. 3). rPRF-stimulated RB6-8C5 depleted mice also produced significant levels of TNF-α (Fig. S4).This suggests that the cell population required for recognition of profilin and production of MCP-1 is not subject to depletion by RB6-8C5 MAb.

Because RB6-8C5 significantly depletes Ly6G^+^ neutrophils as well as Ly6C^hi^ monocytes, we also depleted mice with the Ly6G specific MAb 1A8 [44] to establish the relative contribution of Ly6G^+^ cells. In contrast to CCR2^−/−^ and RB6-8C5-depleted mice, 1A8-depleted rPRF-stimulated animals were consistently protected against *L. monocytogenes* infection (Fig. 6C). rPRF-stimulation reduced bacterial burdens in the spleens of 1A8-depleted mice by ∼3 logs and in the livers by ∼2.3 logs, although bacterial burdens in the livers of all 1A8 depleted mice were highly variable. This observation along with the fact that rPRF stimulated CCR2^−/−^ mice had a 10-fold reduction in liver bacterial burdens may indicate that neutrophils play a minor role in defense in this organ. rPRF-stimulated 1A8-depleted mice also did not show weight loss in contrast to PBS-stimulated controls which lost significantly more weight (Fig. 6C).

Together, these results indicate that although rPRF stimulates a large influx of Ly6C^int^ LyG^+^ neutrophils into the blood and spleen, these cells are largely dispensable for rPRF induced protection and reduction of bacterial burden in the spleen and liver. While Ly6G^+^ neutrophils may have a small contribution in the liver following rPRF-treatment, CCR2-dependent recruitment of Ly6C^hi^ CCR2^+^ inflammatory monocytes plays the central and essential role in rPRF-induced clearance of *L. monocytogenes.*

## Discussion

In this study we investigated how chronic infection with *T. gondii* protects the rodent host against unrelated pathogens [19], [20], [21], [22], [23], [24], [25]. Because rodents are the primary reservoir for *T. gondii*, elucidating the key ligand/receptor interactions is essential for understanding host defense. Our work identifies TgPRF as a *T. gondii* factor that recruits inflammatory monocytes and demonstrates that stimulation of TLR11 or TLR11/TLR12 heterodimers provides an immunological benefit to a *T. gondii*-infected host against another pathogen. Stimulation with TgPRF results in production of MCP-1 and recruitment of Ly6C^hi^ CCR2^+^ inflammatory monocytes and Ly6G^+^ neutrophils into the blood and spleen, although only Ly6C^hi^ CCR2^+^ inflammatory monocytes and CCR2-signaling are essential to reduce bacterial burdens. These data have significant implications for our understanding of the biology of *T. gondii* infection and the evolutionary maintenance of TLR11 in rodents.

Ly6C^hi^ CCR2^+^ inflammatory monocytes were first identified in *L. monocytogenes* infection, where they differentiate into TipDCs at the sites of bacterial infection and are essential for early control of bacterial replication. Emigration of these cells out of the bone marrow is directed by the chemokines MCP-1 and MCP-3 and their receptor CCR2 [12], [16]. Accordingly, CCR2 mice have diminished numbers of TipDCs in the spleen and are highly susceptible to *L. monocytogenes* infection [15]. Ly6C^hi^ monocytes have also been implicated in defense against many other pathogens, including *T. gondii*. In both oral and parenteral *T. gondii* inoculation, Gr-1^+^ Ly6C^+^ monocytes are recruited to sites of infection and are critical for acute survival [8], [9], [10], [11]. These cells have been shown to produce TNF-α and iNOS, and their emigration is dependent on MCP-1 and CCR2 consistent with inflammatory monocytes or TipDC precursor populations, although interestingly they do not appear to acquire CD11c [8], [9], [10], [11]. MCP-1^−/−^ and CCR2^−/−^ mice, which fail to recruit inflammatory monocytes, have enhanced mortality, greater parasite burdens, and die of pathological inflammation and intestinal necrosis [8], [9], [10], [11]. These studies show that Ly6C^+^ monocytes are essential for early control of *T. gondii* replication and to prevent immune pathology. However, the parasite factors that elicit Ly6C^+^ monocytes had not been identified. Here we identify TgPRF as a mechanism by which *T. gondii* can elicit emigration of a Ly6C^hi^ CCR2^+^ inflammatory monocyte population and show that these cells are required for TgPRF to confer resistance to *L. monocytogenes* infection. In this study stimulation by TgPRF was associated with production of the CCR2 ligand MCP-1 but we did not examine production of other notable CCR2 ligands such as MCP-3. Presumably MCP-3 is also involved in CCR2 dependent inflammatory monocyte recruitment during *T. gondii* infection as mortality and defects in monocyte recruitment and are less severe in MCP-1^−/−^ than CCR2^−/−^ mice [10], although no specific studies have addressed the role of this chemokine. We also did not determine if TgPRF recruited monocytes acquire CD11c or differentiate into TipDCs during the context of *L. monocytogenes* infection.

Stimulation with TgPRF also results in trafficking of Ly6C^int^ Ly6G^+^ neutrophils into the blood and spleen. Early work suggested that neutrophils were the major cells responsible for controlling the early growth and dissemination of *L. monocytogenes* [45], [46]. These observations were based mainly on studies using an anti-granulocyte receptor-1 (Gr-1) MAb, which is now known to recognize both neutrophils (Ly6C^int^ Ly6G^+^) and non-neutrophil Ly6C^+^ cells, including subsets of monocytes, macrophages, DCs and lymphocytes [43]. Recent work has suggested that Ly6G^+^ neutrophils are largely dispensable for innate defenses [47] while others have shown that these cells contribute to significant anti-listerial defenses in the liver [48], [49]. Consistent with these findings, we observed that 1A8 depleted mice are slightly more susceptible to *L. monocytogenes* than WT mice (lethal dose 1×10^4^ versus 6×10^4^ CFU), although not as susceptible as CCR2^−/−^ (8×10^3^ CFU) or RB6-8C5 depleted animals (200 CFU). The fact that rPRF-stimulated 1A8 depleted mice are resistant to *L. monocytogenes* infection demonstrates that rPRF-recruited Ly6G^+^ neutrophils are dispensable for TgPRF-induced protection. Rather, Ly6C^hi^ CCR2^+^ inflammatory monocytes and TipDCs play the predominant role in TgPRF-mediated defenses.

There are several mechanisms by which TgPRF recruited monocytes may contribute to early control of *L. monocytogenes* and that could also account for the requirement for IFN-γ. First, inflammatory monocytes may be directly bactericidal. Inflammatory monocytes recruited to the peritoneal cavity during *T. gondii* infection express iNOS and have enhanced parasite killing *in vitro* [11], so it is reasonable to infer that TgPRF recruited monocytes would display enhanced activity against *L. monocytogenes* as well. However, rPRF treatment effectively reduced bacterial burdens in *L. monocytogenes* infected iNOS deficient mice (data not shown) suggesting that NO production is unlikely to be a primary mechanism of killing. The impaired protection we observed in IFN-γ^−/−^ mice could be due to generalized defects in antimicrobial effector mechanisms dependent on IFN-γ that stimulation with TgPRF cannot overcome or because the development of Ly6C^hi^ inflammatory monocytes into TipDCs and inflammatory DCs during *L. monocytogenes* and *T. gondii* infections is largely dependent on NK1.1^+^ cell derived IFN-γ [50], [51].

Noncognate antigen driven proliferation and activation of memory T cells and innate NK cells could also mediate a degree of resistance dependent on IFN-γ and explain the IFN-γ dependence of TgPRF induced protection. Memory T cells can proliferate, produce IFN-γ and acquire effector cell functions during bacterial infection, which contributes to IFN-γ mediated defenses [52], [53], [54]. Activation is driven by IL-15 and IL-18 production by inflammatory monocytes and CD8α^+^ DCs, dependent on inflammasome activation, type I interferon and TLR priming [53], [54]. TgPRF could contribute to induction of noncognate memory T cell responses by increases in the number of inflammatory monocytes or serving as the TLR-based priming signal via stimulation of TLR11. Activation of transferred IFN-γ sufficient memory T cells mediated a ∼100-fold reduction in *L. monocytogenes* bacterial burden in IFN-γ-/- mice, but only modest 3-fold reduction in mice with intact IFN-γ responses [52], [53], [54]. Thus, it is unclear if the 2,500- to 30,000-fold reductions we describe in *T. gondii* infected or rPRF stimulated IFN-γ sufficient mice can be entirely attributed to cognate antigen independent induction of IFN-γ by memory T cells. Inflammatory monocytes can also induce IFN-γ production by NK cells [53]. Along these lines, TgPRF has been shown to stimulate IFN-γ production by NK1.1^+^ cells [6] and NK1.1^+^ derived IFN-γ is required for *T. gondii* induced protection against influenza [25]. In our model however, NK1.1^+^ cells do not appear to be essential for TgPRF mediated defenses against *L. monocytogenes.* The increased importance of NK cells in defense against influenza may be attributable to the comparatively increased role of NK cells in viral infections and killing of virus infected cells.

All of these mechanisms are unable to fully account for the fact that stimulation with rPRF was able to reduce *L. monocytogenes* bacterial burdens in Rag1^−/−^ NK1.1 depleted mice. It is possible that in the absence of T and NK cells, alternative mechanisms leading to production of IFN-γ may be induced. Neutrophils could be an important source of IFN-γ independent of T and NK cells in our model. Recent evidence has clearly shown that IFN-γ producing neutrophils are present in the peritoneal cavity during *T. gondii* infection of WT and TLR11^−/−^ mice and are a biologically relevant source of IFN-γ [55]. Neutrophil derived IFN-γ is produced independent of IL-12 [55], which is consistent with our results showing that neither T cells, NK1.1^+^ cells, nor IL-12 are required for TgPRF-induced resistance to *L. monocytogenes*. The fact that stimulation with TgPRF elicited a significant number of neutrophils suggests that IFN-γ producing neutrophils could provide a relevant source of non NK1.1^+^ derived IFN-γ in our model. Future studies will determine if TgPRF elicits these IFN-γ producing neutrophils during *T. gondii* infection of mice.

The identification of TgPRF as a *T. gondii* factor that elicits Ly6C^hi^ inflammatory monocytes and neutrophils is especially important for our understanding of *T. gondii* infection given that humans presumably lack functional TLR11 and TLR12 receptors for TgPRF, yet inflammatory monocytes are critical for innate defenses against *T. gondii*. In mice, Ly6C^+^ and Gr-1^+^ cells are recruited to sites of *T. gondii* infection in a CCR2 dependent manner and produce TNF-α and iNOS [8], [9], [10], [11]. CCR2^−/−^ and MCP1^−/−^ mice fail to control parasite replication and are highly susceptible to both oral and parenteral *T. gondii* infection [8], [9], [10]. Lack of inflammatory monocytes is associated with severe inflammation at the sites of *T. gondii* infection, including increased numbers of neutrophils, intestinal necrosis and CNS pathology [8], [9], [10]. However, the beneficial versus detrimental role of TgPRF is unclear. Similar to mice lacking inflammatory monocytes, lack of TLR11 during systemic *T. gondii* infection is associated with inappropriate inflammation [56], which suggests a role for TgPRF recruited monocytes in the regulation of systemic immunopathological responses. In contrast, recognition of TgPRF is detrimental during oral *T. gondii* infection, likely because gut commensal bacteria stimulate anti-parasitic immune responses [57]. WT, but not TLR11^−/−^, mice develop acute ileitis and liver pathology suggesting that additional parasite or bacterial factors may be sufficient to direct recruitment of inflammatory monocytes in the absence of TLR11, but concurrent stimulation by gut microbes, TgPRF and other *T. gondii* molecules promotes overwhelming pathological inflammation. The detrimental effects of TgPRF recognition may also be due to TgPRF mediated recruitment of neutrophils, which lead to mucosal pathology [8], [9], [58] and contribute to parasite spread within the intestine [59]. Even so our work presented here shows that recognition of TgPRF and subsequent recruitment of inflammatory monocytes provides a host the benefit of innate defense against an unrelated pathogen. It is possible that carriage of *T. gondii* may have driven the maintenance of TLR11 specifically in rodent hosts due to this property, and that the interaction of TgPRF with TLR11 or TLR11/TLR12 heterodimers may be critical for this beneficial host-microbe interaction.

Other microbes are known to confer symbiotic-like protection against unrelated pathogens. Latent infection with the murine γ-herpesvirus MHV68 and the β-herpes virus MCMV conferred protection against the bacterial pathogens *L. monocytogenes* and *Yersinia pestis* [60]. Protection resulted in increased survival and correlated with 100-fold reductions in *L. monocytogenes* burdens in the spleen and liver, similar to the results we observed with chronic infection by *T. gondii* and stimulation with TgPRF. MHV68 infection also confers enhanced resistance to influenza A virus infection associated decreased viral titers, similar to previous results we reported for *T. gondii* infection [25], [61]. γHV68-induced protection against both *L. monocytogenes* and influenza was associated with elevated IFN-γ and increased numbers of activated macrophages with enhanced antibacterial activity [60], [61]. These results suggest that herpes virus and *T. gondii* exploit similar mechanisms to enhance antibacterial innate immunity.

Inflammatory monocytes and TipDCs play key roles in defense against several other pathogens. Ly6C^+^ monocytes are recruited in CCR2 dependent manner and help initiate protective T cell responses following infection with *Mycobacterium tuberculosis*, *Leishmania major*, and *Cryptococcus neoformans* [14]. The importance of Ly6C^+^ monocytes against *C. neoformans* infection may explain prior observations that chronic *T. gondii* infection confers a survival benefit during co-infection with this pathogen [21]. Ly6C^+^ monocytes have been shown to reduce *Plasmodium chabaudi* circulating parasitemia in a mouse model of malaria and to enhance clearance of West Nile Virus [14]. Inflammatory monocytes and TipDCs may play a more limited or even detrimental role in other infections. TNF-α and nitric oxide produced by TipDCs contribute to tissue injury and liver necrosis during infection with *Trypanosoma brucei* [14]. TipDCs are recruited to the bladder via CCR2 during uropathogenic *E. coli* infection but are dispensable for bacterial clearance [62]. Future studies will examine the role of TgPRF recruited inflammatory monocytes during *T. gondii* and other infections.

## Materials and Methods

### Ethics statement

Animals were housed under conventional, specific-pathogen-free conditions and were treated in compliance with guidelines set by the Institutional Animal Care and Use Committee of the University of Wisconsin School of Medicine and Public Health (IACUC), according to IACUC approved protocol number M01545. This protocol adheres to the regulations and guidelines set by the National Research Council. The University of Wisconsin is accredited by the International Association for Assessment and Accreditation of Laboratory Animal Care.

### Mice

Unless indicated otherwise, all mice used in this study were on a C57BL/6 background and used at 6–8 weeks of age. Wild-type (WT) mice were purchased from National Cancer Institute – Harlan, Frederick, MD. IL-12Rβ1^−/−^ (002984, B6.129S1-*Il2rb1^tm1Jm^*/J), IFN-γ^−/−^ (002287, B6.129S7-*Ifng^tm1Ts^*/J), Rag1^−/−^ (002216, B6.129S7-*Rag1^tm1Mom^*/J), CCR2^−/−^ (004999, B6.129S4-*Ccr2^tm1Ifc^*/J) mice were purchased from Jackson Laboratory (Bar Harbor, ME). TLR11^−/−^ mice were a generous gift from Felix Yarovinsky [5] and were rederived at the University of Wisconsin. A/J mice (National Cancer Institute) were used for protein purification experiments because they more susceptible to *L. monocytogenes* infection than C57BL/6 mice [63], which allowed us to detect subtle changes in bacterial burdens in partially active fractions. All animals were housed and bred under specific pathogen free conditions at an AALAC accredited facility at the University of Wisconsin School of Medicine and Public Health. All experiments were conducted in accordance with an IACUC approved protocol.

### *L. monocytogenes* infections

*L. monocytogenes* strain EGD was a kind gift from C. Czuprynski. Mice were anesthetized with an isofluorane vaporizer connected to an IVIS 200 imaging system (Caliper Life Sciences, Hopkington, MA) then infected via retro-orbital i.v. injection with an appropriate number of bacteria to cause lethal infection as indicated. Animals were monitored daily for clinical signs of disease (ruffled fur, hunched posture, paralysis, etc.) and were euthanized if moribund. At 72 hours post infection, weight loss and bacterial burdens (CFU/g) in the spleen and liver were determined. WT mice were infected with approximately 6×10^4^ CFU (∼6 LD_50_'s), which consistently resulted in death or euthanasia of 100% of control animals. TLR11^−/−^, IL-12Rβ1^−/−^, IFN-γ^−/−^, Rag1^−/−^ NK1.1-depleted, WT RB6-8C5 (Ly6C/Ly6G)-depleted, CCR2^−/−^, and WT 1A8 (Ly6G)-depleted mice were infected with approximately 4×10^4^ CFU, 1×10^4^ CFU, 200 CFU, 8×10^4^ CFU, 200 CFU, 8×10^3^ CFU and 8×10^3^ CFU respectively. These doses were chosen because they resulted in bacterial burdens and weight loss similar to lethally infected WT mice.

### *T. gondii* infection

10 week old WT mice were injected i.p. with 250 tachyzoites of the *T. gondii* strain PruΔ. In order to increase the number of animals that survived greater than 30 days into chronic infection, *T. gondii-*infected and control uninfected mice were all fed a diet containing sulfadiazine (1,365 ppm) and trimethoprim (275ppm) (TD.06596, Harlan Teklad, Madison, WI) from days 9 through 14 post *T. gondii* infection, then returned to a normal diet on day 15 through the duration of the experiment.

### STAg

For experiments in WT mice, soluble *T. gondii* antigens (STAg) was made from sonicated tissue culture grown tachyzoites (4×10^8^/ml) essentially as described previously [25] and typically had a protein concentration of ∼1 mg/ml. For experiments in TLR11^−/−^ mice, double the amount of parasites (8×10^8^/ml) were used.

### Recombinant profilin

Purified recombinant his-tagged *T. gondii* profilin (rPRF) was a kind gift from F. Yarovinsky [5]. rPRF preparations used in this study had endotoxin levels of 6×10^−4^ EU or 1×10^−2^ EU per 100 ng dose (estimated 0.06 and 1 pg endotoxin respectively) as measured by LAL assay (Pierce, Rockford, IL).

### Cytokine analysis

Blood was collected from mice via the lateral tail vein 2 or 24 hours post stimulation with rPRF as indicated. Serum was frozen in aliquots at −80°C and then analyzed using a Mouse Inflammation Cytometric Bead Array kit (BD Biosciences, San Jose, CA) according to the manufacturer's instructions.

### Flow cytometry

Spleens were dissociated by mechanical disruption and digested with collagenase/dispase (20 ug/ml, Roche, Indianapolis, IN) and DNAse I (300 ug/ml, Roche) for 30 min at 37°C and passed through a 70 um cell strainer (BD Biosciences, San Jose, CA). Heparinized blood was collected via cardiac puncture and RBCs were removed by dextran sedimentation. Remaining RBCs were lysed with ammonium chloride. Cells were stained at 4°C in PBS with Live/Dead Violet Fixable Stain kit (Invitrogen, Carlsbad, CA), washed, then stained in PBS with 0.5 mM EDTA, 0.2% BSA, 0.09% azide, and 2% normal rat serum (Jackson ImmunoResearch, West Grove, PA). Anti-mouse CD45 (30-F11) APC, anti-mouse CD11b (M1/70) PerCpCy5.5 (eBioscience, San Diego, CA), anti-mouse Ly6C (AL-21) PE, anti-mouse CCR2 (475301) Fluorescein (R&D Systems, Minneapolis, MN), and anti-mouse Ly6G (1A8) PE-Cy7 antibodies were purchased from BD Biosciences except as indicated. Anti-Rat/Hamster CompBeads (BD Biosciences) were used to set compensation. Data were collected on an LSRII cytometer (BD Biosciences) and analyzed with FlowJo 7.6.1 (TreeStar, Ashland, OR).

### *In vivo* depletions

All antibodies used for depletions were purchased from BioXCell (West Lebanon, NH). To deplete NK cells, mice were treated with the anti-NK1.1 MAb PK136 (250 µg/animal). To deplete both Ly6C^hi^ inflammatory monocytes and Ly6C^int^ Ly6G^+^ neutrophils, mice were treated with anti-Gr-1 MAb RB6-8C5 (250 µg/animal) which recognizes a common epitope shared by Ly6C and Ly6G [43]. To deplete only neutrophils, mice were treated with anti-Ly6G MAb 1A8 (250 µg/animal) which has been shown to deplete neutrophils in the spleen, liver and blood [44], [48], [49]. All depletion treatments were administered in PBS via i.p. injection beginning 48 hours prior to stimulation with rPRF, then continued every 48 (1A8) or 96 (PK136 and RB6-8C5) hours after for the duration of the experiment.

### Statistical analysis

Graphs and statistical analysis were made using Graph Pad Prism (San Diego, CA).Graphs represent means and error bars represent standard deviation except where noted otherwise. Bacterial burden data and cytokine were analyzed with the two-tailed student's t-test, and survival data were analyzed using the Log-rank (Mantel-Cox) method. p-values are represented by asterisks in figures as follows: *p<0.05, **p<0.01, ***p<0.001, and ****p<0.001. We consider all p-values <0.05 to be significant.

## Supporting Information

Figure S1 ***T. gondii*** **profilin is present in an ammonium sulfate precipitation fraction of STAg which reduces bacterial burdens in mice.** (A) STAg was incubated with 5 mM calcium chloride and 100 µg/ml proteinase K for 12 hours at 37°C, followed by addition of 2 mM EGTA for one hour to promote auto-proteolysis of proteinase K, and then incubated at 65°C for 20 minutes (Proteinase K-STAg). A sample of undigested STAg was incubated under the same conditions, except without the addition of proteinase K (STAg). A/J mice (n = 3/group) were stimulated i.v. with either 200 µl STAg (grey), Proteinase K- STAg (white), or PBS (black) 24 hours prior to infection with ∼2×10^3^ CFU of *L. monocytogenes*. A/J mice were used for these experiments because they more susceptible to *L. monocytogenes* infection that C57BL/6 mice [63], and a 200 µl dose of STAg was used because of protein loss, degradation and dilution during purification. At 72 hours post *L. monocytogenes* infection, bacterial burdens of the spleens were quantified. Data shown are the mean ± SD from one of two independent experiments, and were analyzed by ANOVA then compared to PBS-treated group using Dunnett's post-hoc test. *** indicates p<0.001. (B) STAg was subjected to a sequential series of ammonium sulfate (AS) precipitations to separate proteins that remained soluble between 0–30%, 30–45%, 45–60%, or >60% AS. Samples of each fraction were combined to create a positive control. A/J mice (n = 3/group) were stimulated i.v. with either 1 µl of STAg, 1.64 µl of individual or pooled fractions using larger volumes to account for estimated volume increase and protein loss, or PBS 24 hours prior to infection with ∼2×10^3^ CFU of *L. monocytogenes*. At 72 hours post *L. monocytogenes* infection, bacterial burdens of the spleens were quantified. Data shown are the mean ± SD from one of two independent experiments, and were analyzed by ANOVA then compared to PBS-treated group using Dunnett's post-hoc test. *** indicates p<0.001. (C) Western blot of AS fractions from (B) with TgPRF anti-serum.
(EPS)

Figure S2 **Stimulation of TLR11^−/−^ mice with STAg.** TLR11^−/−^ mice (n = 7/group) were stimulated i.v. with 200 µl of double concentration STAg made from parasites at 8×10^8^/ml (dark grey) or PBS (black) 4 hours prior to infection with ∼4×10^4^ CFU of *L. monocytogenes*. At 72 hours post *L. monocytogenes* infection, bacterial burdens of the spleens and liver and percent weight change as compared to immediately prior to infection were quantified. Data shown are the mean ± SD from two pooled independent experiments. * indicates p<0.05.
(EPS)

Figure S3 **Inflammatory monocyte and neutrophil recruitment in response to TgPRF is dependent on TLR11.** Nine month old TLR11^−/−^ male mice (n = 2/group) were stimulated i.v. with 100 ng rPRF (grey) or PBS (black), then blood (A) and spleen (B) cells were collected 4 hours later and the percentage of Ly6C^hi^ inflammatory monocytes (red elliptical gate) and Ly6C^int^ neutrophils (blue square gate) was measured by flow cytometry. Gating and analysis was conducted on singlet, live, CD45^+^ cells. A representative plot for each analysis is shown, and the data shown are the mean ± SD.
(EPS)

Figure S4 **RB6-8C5 depleted mice respond to TgPRF by producing IL-12, MCP-1 and TNF-α.** Mice depleted with anti-Gr-1 (Ly6c/Ly6G) MAb RB6-8C5 (n = 3–4/group) were stimulated i.v. with 100 ng rPRF (grey) or PBS (black). Serum was collected 2 hours later and assayed for cytokine levels by cytometric bead array. Data shown are the mean ± SEM from one experiment. * indicates p<0.05 and ** indicates p<0.01.
(EPS)
